# 

**Published:** 1891-10

**Authors:** 


### Communications.

---

### A CORRECTION.

---

*Editor American Journal of Dental Scieuce:*

My attention has been called to the fact that the name of the Dental Department of the National University of Washington, D. C., does not appear in the "List of Colleges which the National Association of Dental Examiners recommends to the various State Boards to accept as reputable." In justice to that College, I would state that the omission is an error, the correction of which the State Boards will please take notice.

FRED. A. LEVY, D. D. S.,
*Secretary N. A. D. E.*

---

### MARYLAND STATE DENTAL ASSOCIATION.

The annual meeting of the Maryland State Dental Association will be held May 9, 10 and 11th 1892, instead of in December, 1891, as previously announced.

F. F. DREW, D. D. S.,
*Cor. Secretary.*

---

### Monthly Summary.

### To Readers and Correspondents.

Communications intended for publication, and exchange journals and papers, should be addressed to the Editor of The American Journal of Dental Science, No. 845 N. Eutaw St. Baltimore, Md. All letters relating to the business of the Journal, or containing cash remittances, to be directed to Messrs. Snowden & Cowman, No. 9 W. Fayette Street, Baltimore, Md. Articles for publication should be received by the Editor at least two weeks previous to the first of the month on which the number in which it is designed for them to appear, is issued.

☞ The American Journal of Dental Science will contain fifty pages of reading matter in each number, making a volume of about Six Hundred pages,

EXCLUSIVE OF ADVERTISEMENTS

